# Biotransformation of *Dioscorea nipponica* by Rat Intestinal Microflora and Cardioprotective Effects of Diosgenin

**DOI:** 10.1155/2017/4176518

**Published:** 2017-09-20

**Authors:** Jia-Fu Feng, Yi-Na Tang, Hong Ji, Zhan-Gang Xiao, Lin Zhu, Tao Yi

**Affiliations:** ^1^Department of Pharmaceutical Science, Leshan Vocational & Technical College, Leshan 614000, China; ^2^School of Chinese Medicine, Hong Kong Baptist University, Hong Kong Special Administrative Region, China; ^3^Sichuan Academy of Chinese Medicine Sciences, Chengdu 610041, China; ^4^School of Pharmaceutical Science, Guangzhou Medical University, Guangzhou 511436, China; ^5^Laboratory of Molecular Pharmacology, Department of Pharmacology, School of Pharmacy, Southwest Medical University, Luzhou, Sichuan 646000, China; ^6^Shenzhen Research Institute, The Chinese University of Hong Kong, Shenzhen 518057, China; ^7^Institute of Research and Continuing Education (Shenzhen), Hong Kong Baptist University, Shenzhen 518057, China

## Abstract

Studying the biotransformation of natural products by intestinal microflora is an important approach to understanding how and why some medicines—particularly natural medicines—work. In many cases, the active components are generated by metabolic activation. This is critical for drug research and development. As a means to explore the therapeutic mechanism of *Dioscorea nipponica* (DN), a medicinal plant used to treat myocardial ischemia (MI), metabolites generated by intestinal microflora from DN were identified, and the cardioprotective efficacy of these metabolites was evaluated. Our results demonstrate that diosgenin is the main metabolite produced by rat intestinal microflora from DN. Further, our results show that diosgenin protects the myocardium against ischemic insult through increasing enzymatic and nonenzymatic antioxidant levels *in vivo* and by decreasing oxidative stress damage. These mechanisms explain the clinical efficacy of DN as an anti-MI drug.

## 1. Introduction

Ischemic heart disease (IHD) is a significant threat to human health, leading to high morbidity and mortality in the Western world, even in China. It is estimated by the World Health Organization that IHD will be the leading cause of death in the world in the coming decades [1]. Recently, there has been a growing interest in establishing the therapeutic potentials of medicinal plants against IHD. For instance, total peony glycosides from Radix Paeoniae rubrae and cinnamic acid and cinnamic aldehyde from *Cinnamomum cassia* have been evaluated for their protective effect against isoprenaline- (ISO-) induced myocardial ischemia in rats [2, 3]. In particular, it is noteworthy that bioactive steroidal saponins from the medicinal plant *Dioscorea nipponica* (DN) have been successfully developed as effective herbal medicines by the pharmaceutical industry for treating IHD. These herbal medicines developed from DN have been in use since the 1970s; they include “Polysponin” approved by the former Soviet Union's Ministry of Health, and Diosconin Tablet and Di'ao Xinxuekang Capsule produced in China and indicated for myocardial ischemia or angina pectoris.

In the previous study, authors of the present report established a mixed microscopic method for differentiating DN from several *Dioscorea* species in order to ensure the authentic origin of DN during herb collection [4] and later demonstrated that major constituents of DN include *Dioscorea* saponins but contained no free diosgenin [5, 6] and that DN mediates a cardioprotective effect [7]. These findings notwithstanding, the active components and therapeutic mechanism of DN have not been fully characterized. The constituents in DN in their native forms as expressed by plant tissues may be prodrugs; the metabolites, of which, mediate therapeutic effects. Thus, further research is to better characterize their bioactive properties. Such work is anticipated to yield improved insight into clinical use of DN. Our recent research further showed that diosgenin, as a main metabolite from DN, was found and quantified in the plasma from the experimental rat group orally receiving DN [8]. Thus, we hypothesized that diosgenin is a bioactive metabolite related to the antimyocardial ischemia (MI) activity of DN. Diosgenin exerts diverse bioactivities, but most of its pharmacological actions are related to the management of cardiovascular disorders, such as lowering plasma total cholesterol and antithrombotic activity [9]. It is reported that *Dioscorea bulbifera* extract, which is similar to the DN extract that both are rich in diosgenin, improves vascular function by superoxide dismutase (SOD) and catalase (CAT) activity. Thus, it is worthwhile further exploring the anti-MI activity of diosgenin [10].

Recently, identification of metabolites involved in the biotransformation of phytochemicals by intestinal microflora has been suggested as a potentially effective means to determine which compounds are active in living systems and to gain a better understanding of how herbs affect biological processes [11, 12]. To verify our hypothesis, this follow-up study aimed, first, to determine if diosgenin was in fact a metabolite of DN through organ-specific biotransformation by intestinal microflora and, second, to validate the cardioprotective effects of the screened diosgenin using an ISO-induced myocardial ischemia model in rats. The findings of the present study provide a basis for understanding the metabolism of DN, including the identity and mode of action of its active components. The present research supports the use of DN and diosgenin in the clinical management of IHD.

## 2. Materials and Methods

### 2.1. Chemicals and Reagents

General anaerobic medium broth (GAM broth), vitamin K1, and hematin chloride were purchased from Shanghai Kayon Biological Technology Co. Ltd. (Shanghai, China). Analytical grade ethanol purchased from the Merck (Darmstadt, Germany) was used for the extraction of DN samples. Water was purified using a Milli-Q water system (Millipore; Bedford, MA, USA). Acetonitrile (RCI Lab-Scan, Bangkok, Thailand) and methanol (RCI Lab-Scan, Bangkok, Thailand) were used as the mobile phase for analysis. Formic acid (Sigma-Aldrich, USA) was added to the mobile phase for analysis. Isoprenaline was purchased from Sigma (St. Louis, USA). Test kits for SOD, CAT, GPx, T-AOC, and MDA were all purchased from Nanjing Jiancheng Biotechnology Institute (Nanjing, China). Other reagents were of analytical purity.

**Figure 1:**
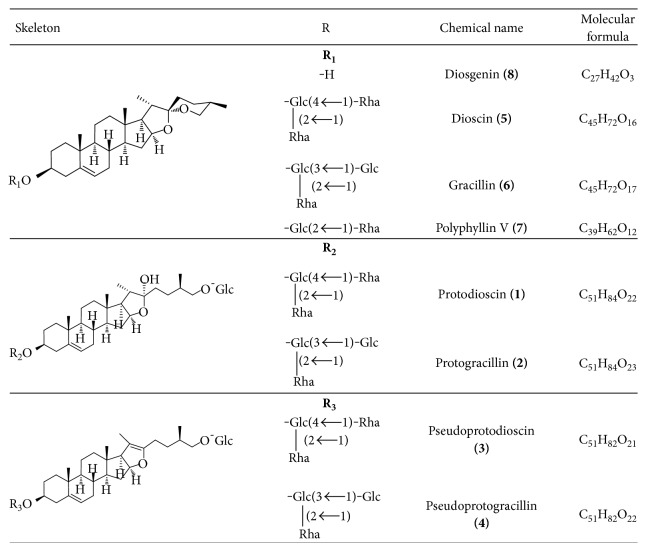
Chemical structures of constituents identified in *Dioscorea nipponica*.

Standards (purity 98%) of protodioscin, dioscin, gracillin, diosgenin, protogracillin, and polyphyllin V were purchased from Phytomarker Ltd., Tianjin. Pseudoprotodioscin was provided by National Institute for Food and Drug Control (Beijing, China). The chemical structures of standards are shown in Figure 1.

### 2.2. Preparation of DN Extract

The rhizomes of DN were collected from the cultivation base in the city of Lingbao in Henan Province, China. All crude drugs were of high quality and authenticated by Dr. Tao Yi, School of Chinese Medicine, Hong Kong Baptist University. Corresponding voucher specimens (number DN-01) were deposited in the Chinese Medicines Center, Hong Kong Baptist University.

The DN samples were dried at 60°C and then pulverized into powder. The powder of DN (200 g) was extracted in an ultrasonic bath with 1000 mL 80% ethanol at room temperature for 1 h. The operation was repeated twice. The combined extracts were evaporated to remove ethanol at reduced pressure in a rotary evaporator (50°C) and then were lyophilized with a freeze-drying system. DN extract (26.1 g, yield 13.05%, *w*/*w*) was thus obtained. The DN extract of 0.1 g was diluted in 10 mL sterile water, filtered through a 0.22 *μ*m pore-sized filter (Millipore, type GV). The filtrate was collected in a sterile tube. These tubes were used as *in vitro* biotransformation vessels. The dried extracts and diosgenin were suspended in 1% (*w*/*v*) aqueous carboxyl methylcellulose for administration to animals.

### 2.3. Preparation of Rat Fecal Samples

Fresh rat feces were immediately collected from ten healthy male Sprague Dawley rats (220–250 g). Samples were collected and mixed, on site, on the day of the experiment and were used immediately. Fecal slurries were prepared by mixing fresh feces samples with autoclaved PBS (0.1 M, pH 7.2) to yield 10% (*w*/*v*) suspensions. The fecal suspensions were filtered through two layers of gauze. The filtered suspensions were then used to inoculate the *in vitro* biotransformation vessels.

### 2.4. In Vitro Biotransformation of DN Extract by Rat Intestinal Microflora

A 30 g GAM broth was dissolved in 1000 mL water (70°C), filtered, while hot, treated with antibacteria process with high pressure (0.15 MPa) and temperature (121°C) for 20 min, and cooled to 45°C. The GAM broth solution was then transferred to an anaerobic chamber (37°C, anaerobic condition), and 1 mg vitamin K1 and 6 mg hematin chloride were dissolved in the solution. Then biotransformation vessels were sterilized and filled with 30 mL of GAM broth solution.

Vessels were inoculated with 3 mL of fecal suspension (10%, *w*/*v*), and then 1 mL of DN extract was added. *In vitro* biotransformation was run under anaerobic conditions for a period of 48 h. Two different control experiments were conducted: (i) incubations of the intestinal microflora in medium lacking the DN extract to monitor metabolites arising from basal metabolism and (ii) incubations of the DN extract in medium without intestinal microflora to monitor changes due to the purely chemical transformation of precursor compounds of the substrate.

The biotransformation mixtures were then prepared according to the method described in the literature [10]. Briefly, the biotransformation mixtures were extracted with 50 mL ethyl acetate three times. The remaining residues were reextracted three times with 50 mL water-saturated n-butanol. The combined n-butanol layers were washed with water three times. Then, the ethyl acetate and n-butanol layers were mixed until homogeneous, concentrated under vacuum, and then diluted to the desired volume with methanol. All solutions were centrifuged at 13,000 ×g for 10 min before being injected for ultra-performance liquid chromatography-mass spectrometry (UPLC-MS) analysis.

### 2.5. UPLC-MS Analysis

An Agilent 1290 series UPLC system (Agilent Technologies, Santa Clara, CA, USA) equipped with a binary pump, an autosampler, and a thermostatically controlled column compartment was used for the chromatographic analysis. A Waters ACQUITY™ BEH C18 column (100 × 2.1 mm, 1.7 *μ*m; Milford, MA, USA) was used for sample separation at 40°C. The mobile phase consisted of 0.1% formic acid in water (A) and 0.1% formic acid in acetonitrile (B) using a gradient program of 0–2 min, 20% B; 2–12 min, 20–28% B; 12–20 min, 28–45% B; 20–35 min, 45–48% B; and 35–46 min, 48–75% B. The sample volume injected was 2 *μ*L, and the solvent flow rate was set at 0.4 mL/min. For mass spectrometric determination, the UPLC system was hyphenated with an ultrahigh definition accurate mass quadrupole time-of-flight mass spectrometry (MS) system (Agilent Technologies G6540A) by a multimode ionization source (G1978-65339) interface. The conditions of MS analysis were optimized as follows: drying gas (N_2_) flow rate, 8.0 L/min; drying gas temperature, 300°C; nebulizer, 45 psi; capillary, 2500 V; and fragmentor voltage, 150 V. Mass spectra were recorded across the range *m*/*z* 100–1700 in both positive and negative modes. All operations and data analysis were controlled by Agilent MassHunter Workstation software version B.04.00.

### 2.6. Animals and Acute Myocardial Ischemia Induced by ISO

Male Sprague Dawley rats weighing 150–200 g were purchased from the Laboratory Animal Services Center, the Chinese University of Hong Kong, Hong Kong. All animals were housed at a room temperature of 23 ± 1°C with a 12 h light/dark cycle. A standard rodent diet and water were provided ad libitum. All experimental protocols were approved by the Committee on the Use of Human & Animal Subjects in Teaching and Research of Hong Kong Baptist University, in accordance with the Animal Ordinance (Department of Health, Hong Kong).

A total of 42 rats were randomly divided into 7 groups: (1) normal control (0.5% *w*/*v* aqueous CMC-Na, i.g.); (2) model group (ISO injection only); (3) positive group (propranolol, 10 mg/kg i.g. for 3 days after ISO injection); (4–6) diosgenin treatment groups. For the diosgenin groups, diosgenin was administered at rates of 20, 40, or 80 mg/kg for 3 days after ISO injection. Dosage was determined from our previous study [7]; (7) DN treatment group was administered with DN extract at 500 mg/kg for 3 days after ISO injection. Diosgenin, DN extract, and propranolol were administered once daily except on the days on which ISO injection was given.

Animals were injected with ISO (1 mg/kg, s.c.) to induce experimental MI twice at an interval of 8 hours on the first day. On the last day of experiment (4th day), the animals were sacrificed. After the rats were anesthetized with diethyl ether, then the blood samples were collected from the femoral arteries of rats. Serum was saved at −80°C following centrifugation at 4°C at 4000 rpm for 20 min.

### 2.7. Histological Examination of Myocardium

Immediately after the sacrifice of the rats, the hearts were removed, washed with iced normal saline, fixed in 10% formalin, and decalcified with formic acid (31.5% formic acid and 13% sodium citrate). The hearts were embedded in paraffin for sectioning by standard histological methods [13]. Sections (4 *μ*m, Leica RM2125, Germany) from the left ventricle were stained with hematoxylin and eosin (H&E) and examined by light microscopy (Leica DMR, Germany) at 200x magnification.

### 2.8. Assays for Biological Activities and Statistical Analysis

Activities of SOD, GPx, CAT, T-AOC, and MDA were measured using kits according to the manufacturers' instructions. Values obtained from the experiments were expressed as the means ± standard deviation (SD). The statistical significance of the differences was assessed by ANOVA followed by post hoc test with LSD method [14, 15]. *P* values less than 0.05 were considered statistically significant.

## 3. Results and Discussion

### 3.1. Choice of Ion Source

Electrospray ionization (ESI) is a soft ionization technique. It is especially useful in producing ions from macromolecules because it overcomes the propensity of these molecules to fragment when ionized. Atmospheric pressure chemical ionization (APCI) is an ionization method used in mass spectrometry which utilizes gas-phase ion-molecule reactions at atmospheric pressure. ESI is today the most widely used ionization technique in chemical and biochemical analyses [16, 17]. However, some analytes (e.g., *Dioscorea* saponin aglycones), for structural and polar reasons, cannot produce enough strong ions with ESI; in these cases, APCI can be used to increase the ion yield. Therefore, in our previous study, ESI and APCI were used to detect saponin glycosides and saponin aglycones, respectively [6]. Due to the need to use different ion sources, each sample had to be analyzed twice, and analysis time was twice as long.

**Figure 2:**
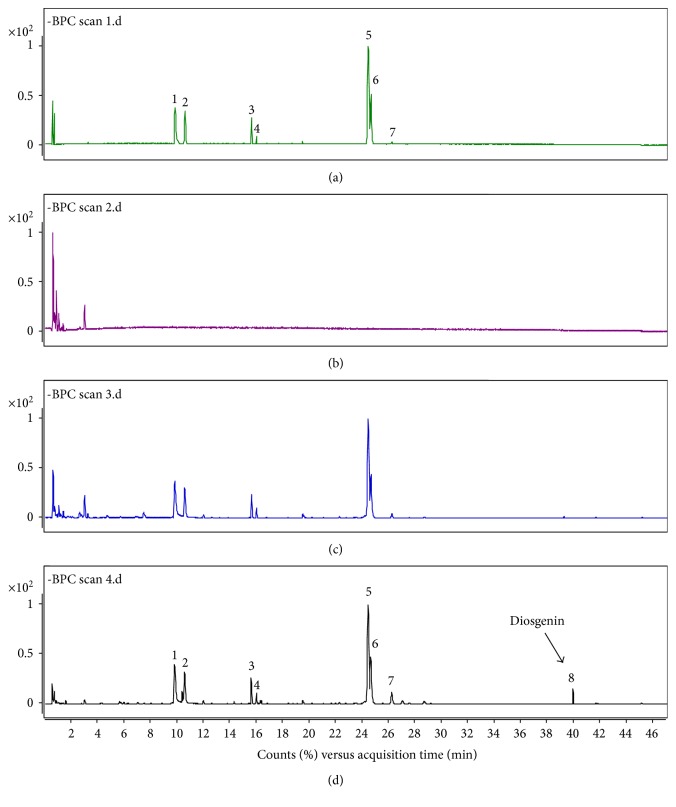
Base peak chromatograms (BPC) of *Dioscorea nipponica* (a), control I using intestinal microflora and medium (b), control II using *D. nipponica* extract and medium (c), *D. nipponica* extract after biotransformed by intestinal microflora and medium (d) by LC–Q-TOF/MS in negative ion mode (1 protodioscin, 2 protogracillin, 3 pseudoprotodioscin, 4 pseudoprotogracillin, 5 dioscin, 6 gracillin, 7 polyphyllin V, and 8 diosgenin).

To solve this problem and save time, an ESI/APCI multimode ionization source was used for LC-MS analysis in the present study. The ESI/APCI multimode source is unique in that it incorporates both ESI and APCI into a single ion source, and it can simultaneously generate ions by ESI and APCI. The main advantages of the multimode source include eliminating the time required to switch ion sources on an instrument and eliminating the need to run samples twice to improve lab productivity [18]. Compared to the previous study, saponin glycosides and saponin aglycones both were ionized and identified in a single run with the help of multimode ionization source. As a result, we find a newly generated peak 8, which corresponds to diosgenin, from the chromatogram of the DN extract incubated with rat intestinal microflora (Figure 2(d)).

### 3.2. Detection of Diosgenin as a Metabolite of DN Extract

The effect of intestinal microflora on drug metabolism has received increasing attention in recent years. Anaerobic bacteria present in the small intestines are quite diverse in species, and different species produce enzymes which are different in functions; it is these enzymes that are also responsible for drug biotransformation in organisms. Incubation of test drugs, particularly phytochemicals, with fresh fecal specimen is a common means for investigating this kind of biotransformation [11, 12].

In the present study, DN extract was tested by this method; the resulting metabolic profile is shown in Figure 2(d). Based on the comparison of samples with standard compounds, seven peaks were unambiguously identified as protodioscin (1), protogracillin (2), pseudoprotodioscin (3), dioscin (5), gracillin (6), polyphyllin V (7), and diosgenin (8) and peak 4 was tentatively identified as pseudoprotogracillin (4) by comparing their *m*/*z* values and MS spectra with the data in the literature [8]. Compared with the identified peaks in Figure 2(a), the new generated peak 8 in Figure 2(d) was attributed to diosgenin. This finding confirmed our hypothesis that intestinal bacteria produce diosgenin from DN extract [8].

### 3.3. Effects of Diosgenin on Myocardial Histology

**Figure 3:**
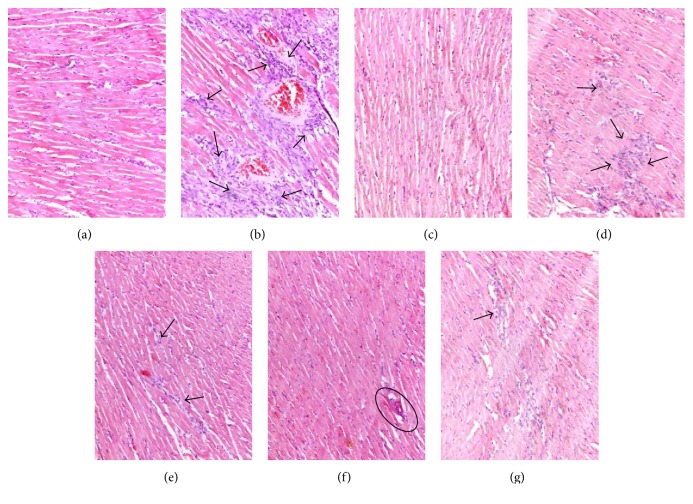
Histopathological changes of myocardial tissue (H&E, ×200). (a) Normal control group showing normal myocardial histology, clear transverse striations, and no inflammatory cell infiltration; (b) ISO group showing swelling of obvious myocardial cells, degeneration, loss of transverse striations, and large numbers of invasive inflammatory cells; (c) POS group (propranolol, 10 mg/kg) showing normal myocardial arrangement, clear transverse striations, and slight inflammatory cell infiltration; (d) diosgenin (40 mg/kg) showing myocardial cell swelling, degeneration, unclear horizontal striations, and large numbers of inflammatory cells; (e) diosgenin (60 mg/kg) showing diminished myocardial cell swelling, unclear horizontal striations, and reduced inflammatory cell infiltration; (f) diosgenin (80 mg/kg) showing normal myocardial arrangement, clear transverse striations, and little few inflammatory cells; (g) DN extract (500 mg/kg) showing normal myocardial arrangement, clear transverse striations, and few invasive inflammatory cells.

Light microscopy of heart tissue sections from normal control rat myocardium showed obvious integrity of myocardial membrane, a normal myofibrillar structure with striations, a branched appearance, and continuity with adjacent myofibrils (Figure 3(a)). Tissue from the rat-given-ISO group revealed loss of transverse striations, marked myocardial cell swelling, large numbers of infiltrating inflammatory cells, and cardiac necrosis (Figure 3(b)). Tissue sections from the rat-given-propranolol-POS group presented approximately normal myofibrillar structure with clear striations and presence of a few inflammatory cells (Figure 3(c)). Low dosage of diosgenin-treated groups showed diminished myocardial cell swelling, unclear transverse striations, and reduced inflammatory cell infiltration compared to the ISO group (Figure 3(d)). Tissues from medium dosage of diosgenin-treated groups revealed less severe histological damage, such as normal myocardial arrangement, clear transverse striations, and few invasive inflammatory cells (Figure 3(e)). Groups treated with high dosage of diosgenin and DN extract exhibited normal, well-preserved cardiac muscle cell histology with no significant damage (Figures 3(f) and 3(g)). These findings demonstrated that diosgenin and DN extract could protect myocardial tissues from pathological damage that would have otherwise occurred from the experimental treatments.

### 3.4. Effects of Diosgenin on SOD, CAT, GPx, T-AOC, and MDA Serum Levels

It is widely accepted that isoprenaline (ISO) injection can readily induce acute MI in rats; it is also widely accepted that antioxidant activity is one of the key mechanisms of anti-MI efficacy [2, 3, 19]. Therefore, it is reasonable to use the ISO model to compare the therapeutic effect of diosgenin with respect to antioxidant activity.

Oxidative stress plays an essential role in the pathogenesis of MI injury. One cause is reactive oxygen species (ROS) resulting from mitochondrial dysfunction via the electron transport chain during MI. The major ROS, such as hydrogen peroxide (H_2_O_2_), superoxide anion (O_2_^−^), and hydroxyl radicals (OH·), are generated during ischemia and particularly during reperfusion [19–21]. However, these potentially deleterious ROS are controlled by external or exogenous antioxidative defense systems which eliminate prooxidants and scavenge free radicals. The most well-known endogenous mitochondrial antioxidant enzyme is SOD, which dismutates superoxide to H_2_O_2_. Other endogenous antioxidant enzymes include catalase and glutathione peroxidase. Exogenous antioxidants are mainly derived from food and herbs. Numerous types of bioactive phytochemicals, such as polyphenolics, glycosides, and steroids, belonging to exogenous antioxidants, have gained attraction in clinical as well as research areas [22–25]. According to published reports describing anti-MI activity of herbal medicine [3, 17, 18], five indices related to the antioxidant activity in the MI model are usually monitored to evaluate the protective effect against ISO-induced injury in cardiomyocytes. These are an indicator of lipid peroxidation, namely, malondialdehyde (MDA); three enzymatic antioxidants, namely, total superoxide dismutases (SOD), catalase (CAT), and glutathione peroxidase (GPx); and an indicator of both nonenzymatic and enzymatic antioxidants, namely, total antioxidant capacity (T-AOC) [26–28]. Therefore, following the widely accepted international rule, SOD, CAT, GPx, T-AOC, and MDA were chosen to assess the anti-MI activity of diosgenin identified in the biotransformation study described above.

**Figure 4:**
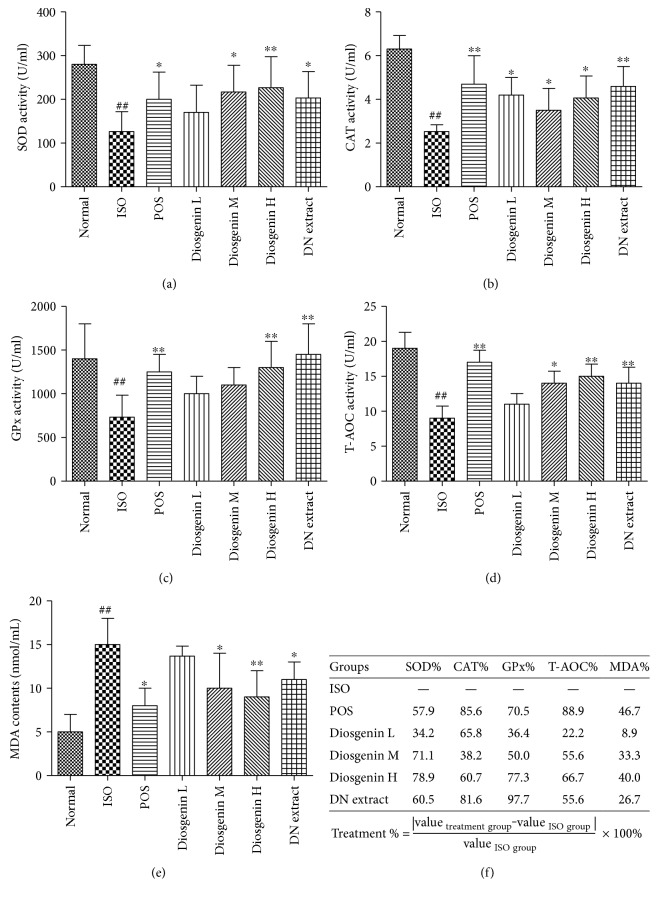
Effects of diosgenin and *Dioscorea nipponica* extract on acute experimental myocardial ischemia and treatment percentage. (i) (a–e) SOD, CAT, GPx, T-AOC, and MDA serum levels. (ii) (f) Treatment percentage of assay markers for each treatment group. Normal: normal control; ISO: model group only injected with isoprenaline; POS: positive control (propranolol, 10 mg/kg); Diosgenin L, Diosgenin M, and Diosgenin H: orally given diosgenin 20, 40, and 80 mg/kg of low, medium and high dose, respectively, after ISO injection; DN extract: orally given *Dioscorea nipponica* extract (500 mg/kg), after ISO injection. Data are expressed as mean ± SD (*n* = 6). ^##^*P* < 0.01 versus normal control; ^∗^*P* < 0.05, ^∗∗^*P* < 0.01 versus ISO group.

Compared with the normal control group, SOD, CAT, GPx, and T-AOC levels in the ISO group decreased significantly (^##^*P* < 0.01), while MDA levels increased significantly (^##^*P* < 0.01) (Figures 4(a), 4(b), 4(c), 4(d), and 4(e)). This meant that our modeling by ISO injection was successful.

Groups administered with different dosages of diosgenin and DN extract exhibited varying degrees of antioxidant activity, as revealed by these five markers (Figures 4(a), 4(b), 4(c), 4(d), and 4(e)). Pathological levels of SOD, CAT, GPx, T-AOC, and MDA in experimental MI rats were almost normalized by diosgenin and DN extract treatments compared with those in the ISO group (^∗∗^*P* < 0.01 or ^∗^*P* < 0.05), except the SOD, GPx, T-AOC, and MDA serum levels in the low dosage diosgenin-treatment groups. These findings suggest that the anti-MI mechanism of diosgenin is related not only to more varieties of enzymatic antioxidant but also to nonenzymatic antioxidants.

Diosgenin administered orally at doses of 20, 40, and 80 mg/kg showed significant dose-dependent increment of the SOD, GPx, and T-AOC serum levels and dose-dependent reduction of the MDA serum levels. The peak treatment effects of diosgenin (78.9% and 77.3%) were recorded with the dose of 80 mg/kg in the SOD level and GPx level, which were higher than those of propranolol with the dose of 10 mg/kg (57.9% and 70.5%), compared with the ISO model group. These results demonstrate that diosgenin has distinct antioxidant properties *in vivo.* Given their known benefits, these antioxidant properties may be responsible for the therapeutic effects of DN in the treatment of MI.

Reduction in oxidative stress caused by ischemia-reperfusion injury is clearly an appropriate countermeasure to the major challenges associated with ischemia. It is worth noting that the antioxidant is not a panacea. A recent study reported that administration of 30 mg/kg/day *β*-carotene could significantly improve heart function of the isolated ischemic/reperfused (I/R) rat hearts through enhancing antioxidant capacity. However, increasing *β*-carotene dosage did not add any cardiovascular benefits. Moreover, the agent may mediate and, indeed, may exacerbate existing I/R pathological mechanisms [29]. Antioxidants play a role of double-edged sword in the occurrence and development of diseases.

## 4. Conclusion

The present study identified the metabolites from DN through analysis of organ-specific biotransformation and validated the cardioprotective effects of the screened metabolites using an isoprenaline-induced myocardial ischemia rat model. The findings of the present study provide evidence that, first, diosgenin is generated from DN by intestinal microflora; second, diosgenin can protect the myocardium against ischemic insult in a dose-dependent manner, almost comparable to the effect of DN extract; and, third, DN's protective effect can be attributed to the increase of enzymatic and nonenzymatic antioxidants (SOD, CAT, GPx, and T-AOC) *in vivo* and to a decrease in lipid peroxidation. These phenomena help explain the clinical efficacy of DN as an anti-MI drug.
